# Staging of Osteochondral Lesions of the Talus: MRI and Cone Beam CT

**DOI:** 10.5334/jbr-btr.1377

**Published:** 2017-12-16

**Authors:** Magdalena Posadzy, Julie Desimpel, Filip Vanhoenacker

**Affiliations:** 1 Radiology Department, W. Dega Orthopaedic and Rehabilitation University Hospital of Karol Marcinkowski University of Medical Sciences Poznan, PL; 2 AZ Sint-Maarten, Duffel-Mechelen, Antwerp University Hospital, BE; 3 AZ Sint-Maarten and University (Hospital) Antwerp/Ghent, BE

**Keywords:** Trauma, Arthrography, Magnetic Resonance Imaging (MRI), Cone Beam CT (CBCT), Musculoskeletal joint, Musculoskeletal bone

## Abstract

Osteochondral lesions (OCL) of the talus involve both articular cartilage and subchondral bone of the talar dome. This term refers to a wide spectrum of pathologies including mild bone marrow contusion as well as severe osteoarthritis resulting from long standing disease. Although Magnetic Resonance Imaging (MRI) at 1.5 Tesla is the leading cross-sectional modality for detection and staging of OCL, lack of spatial resolution hampers accurate assessment of thin articular cartilage. Cone Beam Computed Tomography (CBCT) arthrography is better suited for precise staging of cartilage lesions. The purpose of this pictorial review is to illustrate the strength of each imaging method.

## Introduction

**Figure 1:**
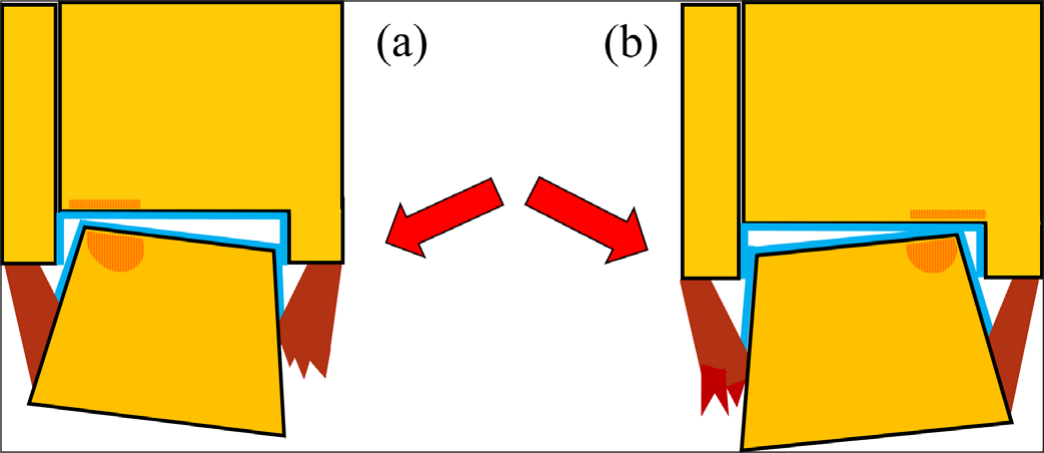
**Location of the OCL according to the mechanism of trauma.** Schematic drawing of talocrural joint injury in pronation **(a)** results in sprain of the medial collateral ligaments (brown) and lateral-sided OCL of the talar dome and/or kissing tibial lesion (orange zones), whereas injury in supination **(b)** causes sprain of the lateral ligaments and medial-sided OCL of the talar dome and/or kissing lesions at the tibia (orange zones). The red arrow indicates the direction of the applied force. The articular cartilage layer of the talocrural joint is indicated in blue.

Osteochondral lesions (OCL) of the talus are defined as any damage involving both articular cartilage and subchondral bone of the talar dome. This term covers a wide spectrum of pathologies including (sub)chondral contusion, osteochondritis dissecans, osteochondral fracture and osteoarthritis resulting from longstanding disease. Subchondral bone involvement can be manifested by bone marrow edema (BME), fracture, sclerosis and/or cyst formation. Cartilage damage may have a variable imaging appearance ranging from a small fissure, a distinct defect, flap formation or delamination. The majority of those lesions occur in active patients and are related to trauma. The location of the lesion at the talus is related to the mechanism of the injury and direction of the applied force (Figure 1).

Osteonecrosis can develop when the lesion’s vascularity is disrupted. The articular surface of the talus is large and its blood supply is critical in the watershed areas [1] explaining an impaired healing process and predisposition to posttraumatic necrosis in those vulnerable areas.

Accurate staging of cartilage lesions is of utmost importance, as this will have a major impact on the treatment strategy and ultimate prognosis. Unstable lesions – if left untreated – predispose for early osteoarthritis. Arthroscopic evaluation of the cartilage is regarded as the gold standard [2], but due to its invasiveness and the need for anesthesia, it should be reserved for preoperatively well-documented cases and combined with surgical treatment procedures. This underscores the value of preoperative imaging.

## Normal Anatomy of Talocrural Joint

**Figure 2:**
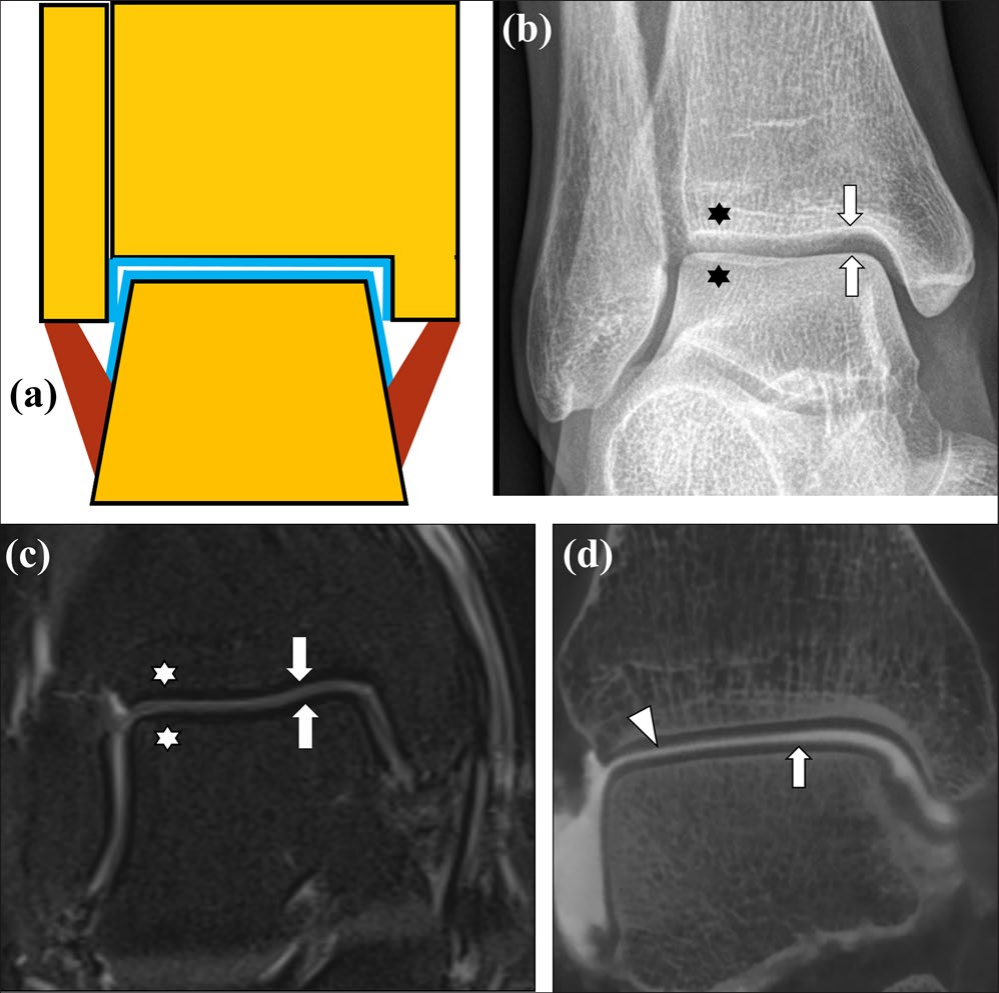
**Anatomy of the talocrural joint.** Schematic drawing of the normal talocrural joint **(a).** Articular cartilage covering the articular surfaces (blue), cortical bone (black), normal bone marrow (yellow) and ligaments (brown). Conventional radiography, Mortise View **(b).** Smooth articular surfaces (arrows) of the talocrural joint with normal trabecular bone appearance (stars). MRI coronal PD fat suppressed image (1.5 Tesla equipment) of the talocrural joint **(c)** with normal appearance of the thin cartilage layer (arrows) of intermediate signal, low signal of subchondral bone and homogenous bone marrow signal (stars). CBCT-Arthrography (CBCT-A) of the talocrural joint, coronal reformatted image **(d)** showing smooth cartilage lining covering the normal subchondral bone of the talus (arrow) and tibia (arrowhead). The presence of intraarticular contrast and high spatial resolution improves visualization of the cartilage surfaces compared to routine MRI on 1.5 Tesla.

Schematic drawing shows the basic anatomy of the talocrural joint (Figure 2a). On plain films, the subchondral bone is seen as a thin layer of compact bone with a smooth surface and a uniform adjacent trabecular bone (Figure 2b). MRI allows for distinguishing normal cartilage from subchondral bone as well as evaluating the adjacent bone marrow, ligaments and other surrounding soft tissues (Figure 2c). Compared to the articular cartilage of the knee, cartilage of the ankle joint is very thin and the spatial resolution of MRI may be insufficient for detection of small lesions. Therefore, for more accurate evaluation of cartilage covering of articular surfaces of the talar dome and distal tibia and fibula, direct arthrographic techniques combined with CT and MRI may be useful (Figure 2d). Recently, Cone Beam Computed Tomography (CBCT) of small joints has been introduced as an alternative technique for Multi Detector CT, combining a very high spatial resolution, low radiation dose and low cost [3].

## Staging of OCL of the Talus

For staging of OCL of the talus several grading systems have been proposed. The first system of classification has been reported by Berndt and Harty in 1959 [4], including four stages based on their radiological appearance. Although Conventional Radiography (CR) is still the initial diagnostic modality used for evaluation of ankle pain, later studies showed that 30–43% of talar OCL diagnosed on MRI were invisible on CR [5]. Later on, this grading system has been modified to computed tomographic evaluation and correlated with arthroscopy, distinguishing cystic lesion of talar dome seen in primary stages with or without communication to the articular surface and detached fragment in more advanced lesions [6]. With the advent of MRI, this grading system was further revised including evaluation of structures invisible on conventional radiology, such as the integrity of the cartilage and presence of BME. A modified grading system has been proposed by Hepple in 1999 [5] and by Dipaola et al. [7], who correlated MR imaging with arthroscopic appearance.

**Figure 3:**
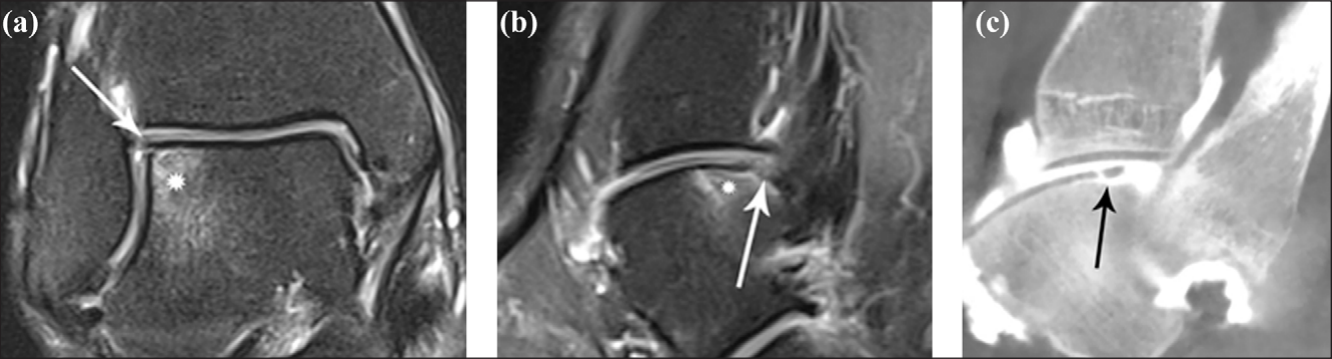
**Example of understaging of the cartilage defect of an OCL on MRI compared to CBCT arthrography.** Coronal fat suppressed T2-WI (intermediate weighting) showing BME (white asterisk) at the lateral corner of the talar dome **(a).** There is slight irregular delineation of the cartilage (white arrow). Sagittal fat suppressed T2-WI (intermediate weighting) showing BME (white asterisk) at the lateral corner of the talar dome **(b).** There is slight irregular delineation of the cartilage (white arrow). On a corresponding sagittal reformatted CBCT-A **(c)**, there is almost complete detachment of the cartilage at the superolateral aspect of the talar dome (black arrow).

**Figure 4:**
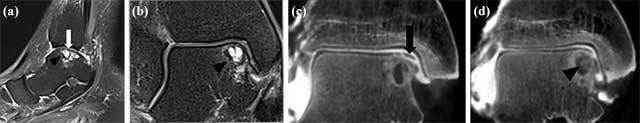
**Example of improved visualization of communication of subchondral cysts with the joint through deep articular cartilage lesions on CBCT arthrography.** Sagittal **(a)** and coronal **(b)** fat suppressed T2-WI showing multilocular subchondral cysts (black arrowheads) at the medial aspect of the talar dome. The cartilage at the talar dome is slightly irregularly delineated (white arrow). Coronal **(c, d)** reformatted CBCT-A clearly shows an extensive cartilage lesion down to bone with adjacent cartilage flap (black arrow). Note also partial filling of the subchondral cyst (black arrowhead) with contrast as an indirect sign of joint communication through a cartilage lesion.

Due to the widespread use of fluid-sensitive sequences on MRI, even subtle foci of BME may be seen adjacent to a cartilage defect, particularly in acute or subacute OCL lesions. On the other hand, although MRI a very useful and sensitive technique for evaluation of the subchondral compartment (showing either BME or cyst formation), the precise depth and extent of the overlying cartilage lesion is often not accurately staged. In adult patients, the depth of the cartilage lesions is often understaged (Figures 3 and 4).

**Figure 5:**
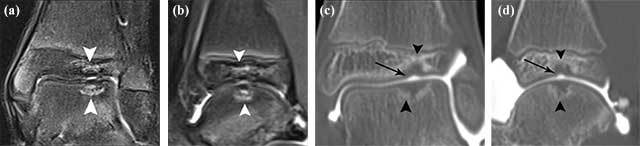
**Example of accurate staging of the status of the articular cartilage in paediatric OCL.** Coronal **(a)** and sagittal **(b)** fat suppressed T2-WI show adjacent kissing areas of bone marrow edema (white arrowheads) at the distal tibia and talar dome. The overlying cartilage is difficult to assess on MRI. Coronal **(c)** and sagittal **(d)** reformatted CBCT-A show a focal bony lesion with peripheral sclerosis in the distal tibia and talus. The overlying cartilage is intact at the talus, whereas there is subtle cartilage lesion at the distal tibia (arrow).

Because the plasticity of the cartilage in children and adolescents is higher than in adults, OCL lesions in young patients are often characterized by isolated subchondral bony changes without overlying cartilage disruption (Figure 5).

**Figure 6:**
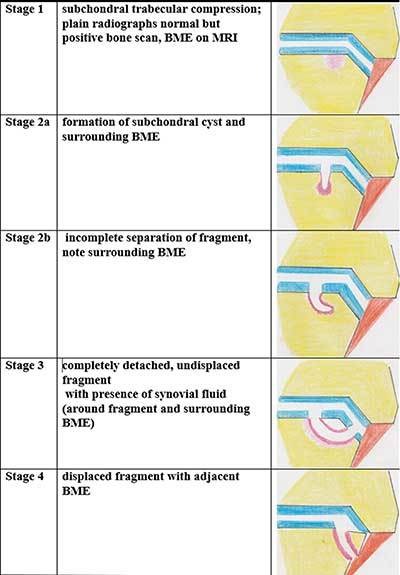
Schematic drawings of OCL classification according to Anderson.

An alternative MRI staging system has been proposed by Mintz [8] et al. in 2003. Nowadays MR staging of OCL on MRI is usually done by the Anderson classification [9], which is another modification of the initial staging system based on plain film evaluation by Berndt and Harty (Figure 6).

**Figure 7:**
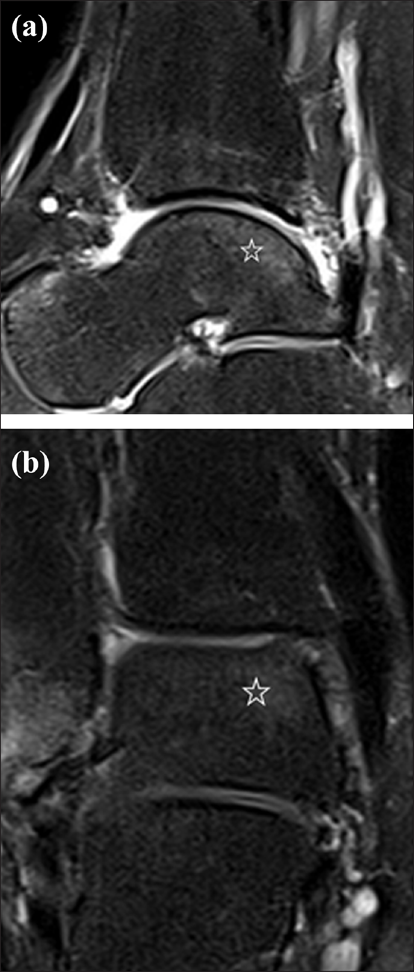
**Stage 1 lesion according to Anderson classification.** Sagittal PD fat suppressed MRI image **(a)** showing BME (star) at the posteromedial part of the talar dome. Coronal PD fat suppressed MRI image **(b)** revealing BME (star) in the posteromedial part of the talar dome.

*Stage 1* lesions are due to bone marrow contusion. MRI is the most sensitive method to depict this stage without any correlating signs on CR or CBCT with injection of intraarticular contrast. Articular cartilage lining remains homogenous without any signal changes (Figure 7).

*Stage 2* refers to partial detachment of OCL with subchondral cyst formation or fissure incompletely separating the lesion from the talar dome.

**Figure 8:**
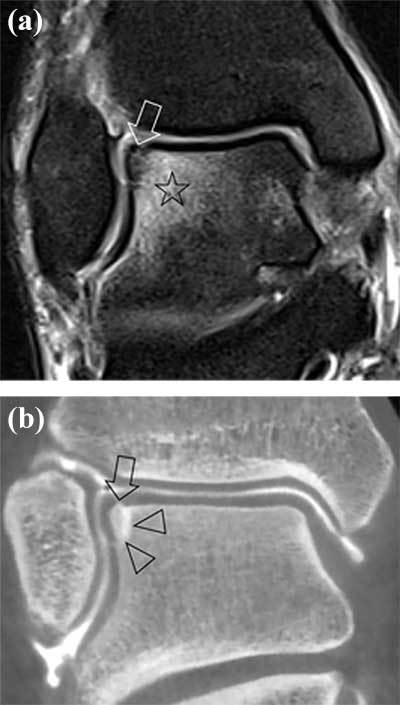
**Stage 3 according to Anderson classification.** Coronal PD fat suppressed coronal image **(a)** showing an OCL fragment completely detached from talar dome without displacement (arrow), bone marrow oedema (star) of adjacent part of the talus. Coronal reformatted CBCT-A **(b)** demonstrates more clearly the OCL fragment (arrow) separated from the adjacent talus by thin rim of surrounding contrast (arrowheads).

In *stage 3* an undisplaced completely separated fragment can be seen on MRI with adjacent BME. On CBCT arthrographic images, the contrast separating the OCL fragment from the talar dome can be evaluated with more confidence (Figure 8).

*Stage 4* consists of a displaced fragment, often accompanied with surrounding bone marrow edema.

**Figure 9:**
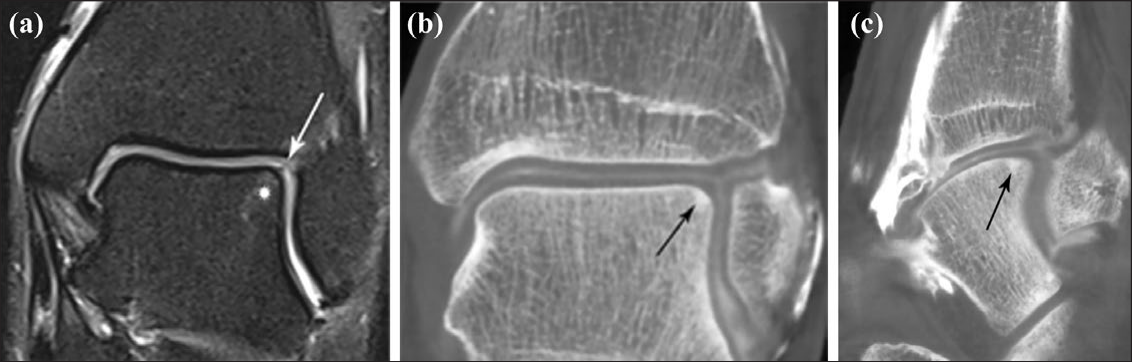
**Down staging of an OCL on CBCT compared to MRI.** Coronal **(a)** fat suppressed T2-WI show adjacent BME (white asterisk) at the superolateral aspect of the talar dome. The overlying cartilage is difficult to assess on MRI but seems to be slightly inhomogeneous (white arrow). Coronal **(b)** and sagittal **(c)** reformatted CBCT-A show subtle subchondral sclerosis (black arrow) at the superolateral aspect of the talar dome, but the overlying cartilage is intact.

**Figure 10:**
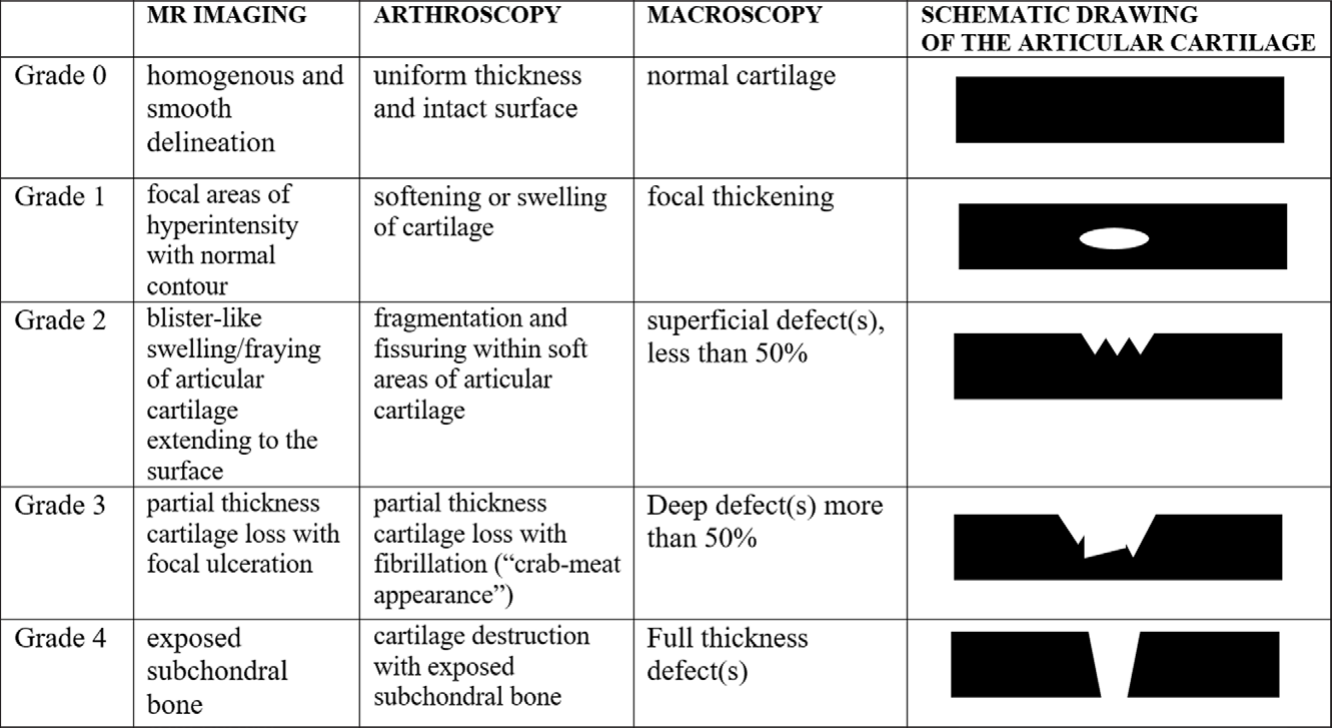
Modification of the Outerbridge classification of cartilage defects.

Useful MR scoring parameters include lesion location, lesion size in 3 planes, subchondral bone marrow edema, subchondral cyst formation and/or sclerosis, status of the overlying cartilage, contour depression of the articular bone plate. Despite the combination of these MR parameters, accurate cartilage evaluation remains often illusive. The main reason for that is the fact that we need images with high spatial resolution to detect early changes of articular cartilage of the ankle joint. MRI sensitivity in detection of OCL of the talus, correlated with arthroscopic correlation, varies according to different studies and has been reported as high as 81% [10]. The accuracy also depends of the strength of the field and is lower on 1.5 Tesla magnets in comparison to 3T [11]. Studies on cadavers performed on CT arthrography [12] showed more accurate cartilage thickness measurements in comparison to standard MRI, which is in line with a superior evaluation of OCL with CT arthrographic techniques [13] (Figure 9). Besides the limitations of MRI in this field, it is still considered the most comprehensive imaging modality of the ankle because of its capability to assess soft tissue and bone marrow abnormalities on a single examination. Moreover, despite several modifications of the staging systems on MRI, not all combination of the degree of involvement of the cartilage and subchondral bone are included and therefore these classification systems remain uncomprehensive, complicated and less valuable for use in daily routine. As MRI is inaccurate for the evaluation of the articular cartilage compartment, further staging with direct arthrographic techniques are often mandatory if an OCL is detected on MRI and in those scenarios in which arthrosopic treatment is considered. Similar to the Outerbridge classification widely used in staging of cartilage lesions of the knee, a modified staging system for evaluation of the depth of cartilage defects with correlation to arthroscopy may be used in the ankle (Figure 10).

Furthermore, cartilage lesions may be isolated (one defect), complex (one lesion with variable depth of the lesion) or multifocal (involving multiple areas of the talus or tibia).

**Figure 11:**
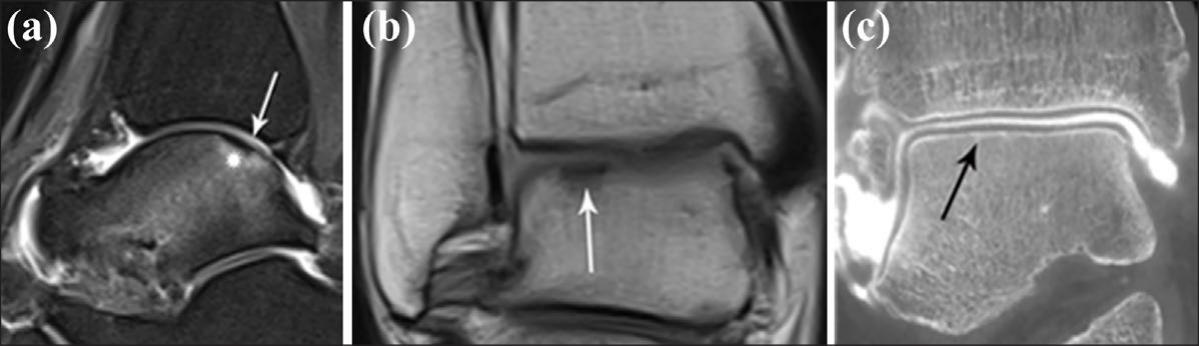
**Alternative diagnosis on CBCT compared to MRI.** Sagittal **(a)** fat suppressed T2-WI show a subchondral band-like area of low signal at the subchondral cortex (white arrow) with surrounding BME (white asterisk). Coronal proton density **(b)** shows focal hypointense thickening of the talar dome (arrow). Coronal reformatted CBCT-A **(c)** barely shows subtle subchondral sclerosis at the superolateral aspect of the talar dome and intact overlying cartilage. Based on the combination of MRI and CBCT findings the diagnosis of a subchondral insufficiency fracture (SIF) was made.

CBCT, which was first introduced for preoperative evaluation of dental implants, is currently also used for musculoskeletal applications. It uses a conical X-ray beam and flat-panel detector collecting all volumetric data in one rotation of the gantry. It combines high spatial resolution, relatively low radiation dose and low equipment cost and is useful for evaluation of trauma of small bones and joints, particularly when there is clinical suspicion for a fracture despite negative plain radiographs [3]. The equipment is designed to perform exams in sitting or supine position and is relatively compact, allowing installation in many radiology departments and private practices. CBCT following intra-articular injection of Iodine contrast (CBCT-Arthrography) may render exquisite detail of the articular cartilage using very thin slices and multiplanar reformation. In addition, the trabecular architecture of subchondral bone is far better visualized on CBCT than on CR. In this regard, CBCT-Arthrography (CBCT-A) may be very promising technique for precise staging of cartilage lesions of the ankle as an alternative for Multi Detector Computed Tomography (MDCT). In particular cases also alternative diagnoses can be made on basis of CBCT (Figure 11).

## Conclusion

Due to its noninvasiveness, absence of radiation exposure and its ability to visualize associated concomitant soft tissue abnormalities, MRI is the initial technique for exclusion/confirmation of an osteochondral lesion of the ankle. Additional CBCT-Arthrography is, however, very useful for more accurate cartilage staging and should be considered in those clinical scenarios where arthroscopic treatment of the lesion is considered.
